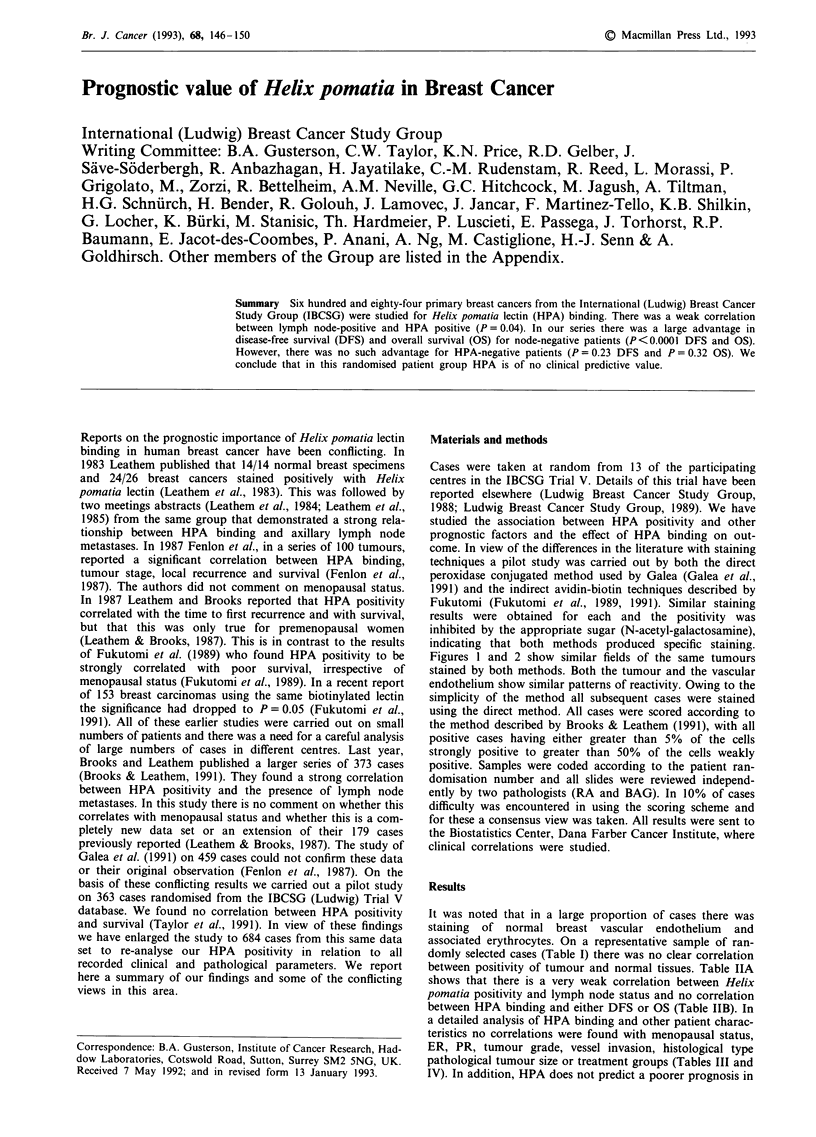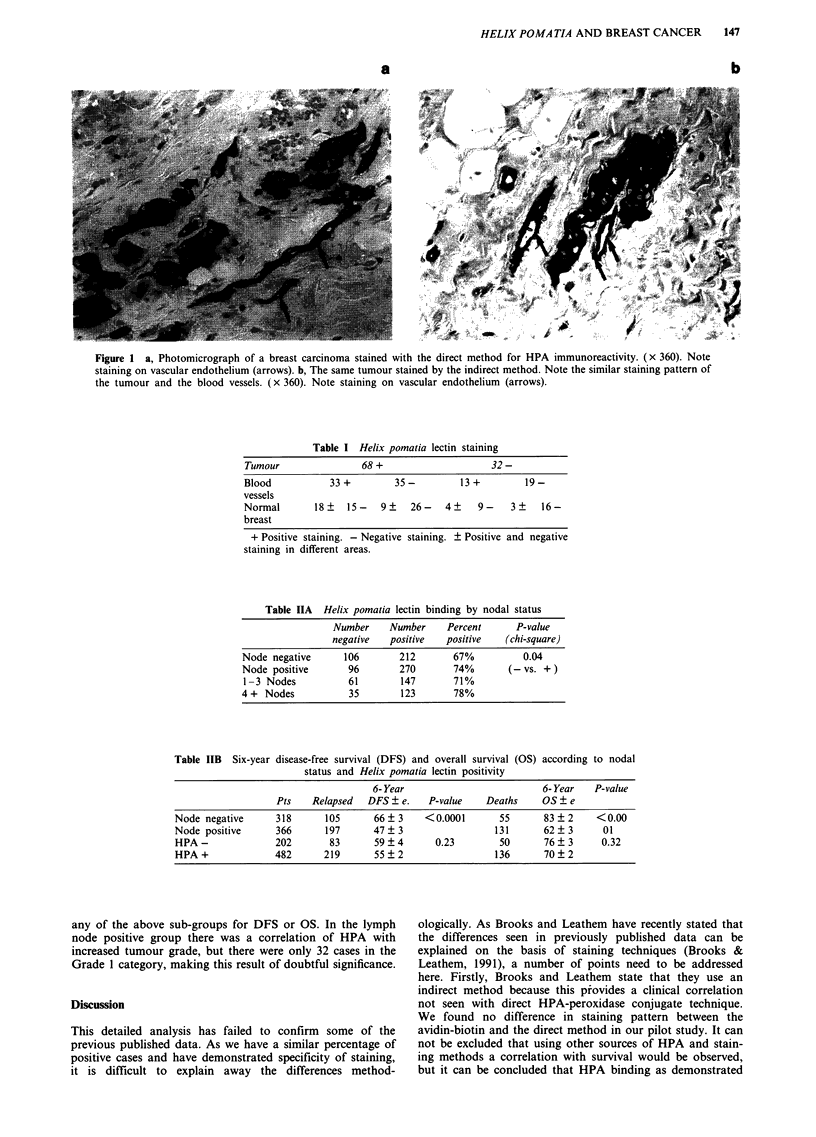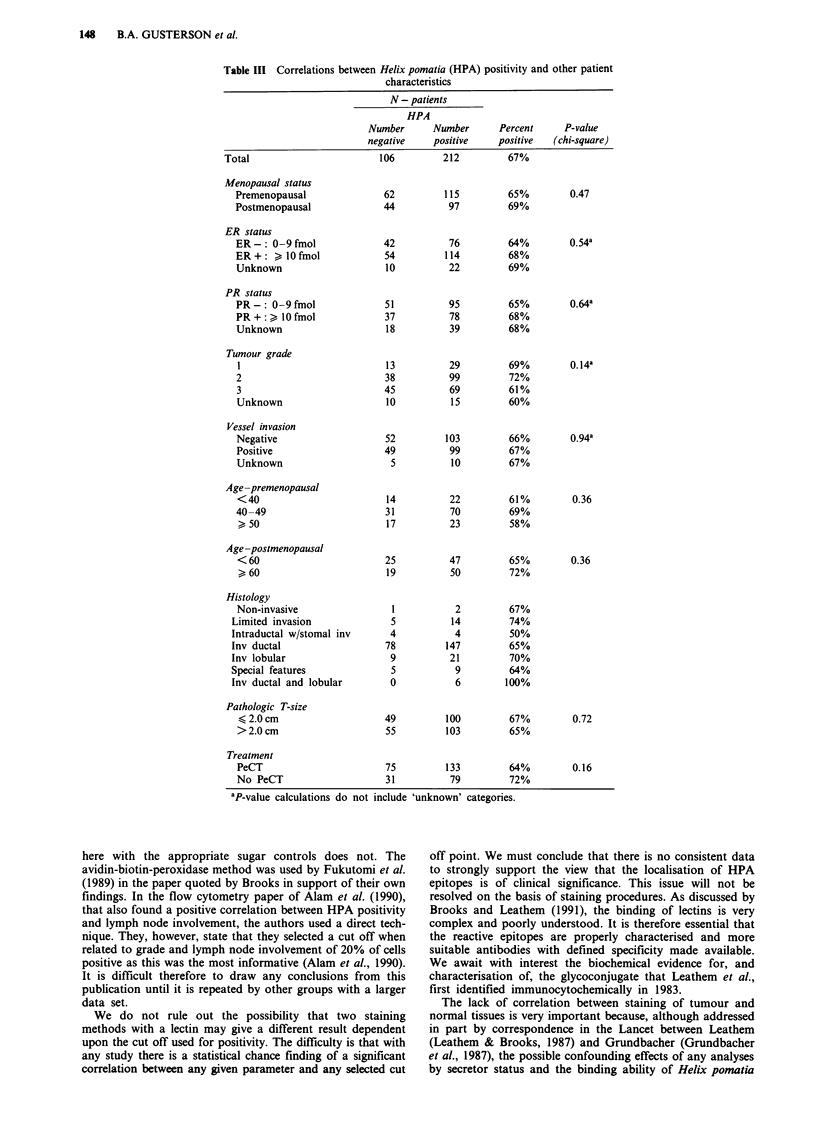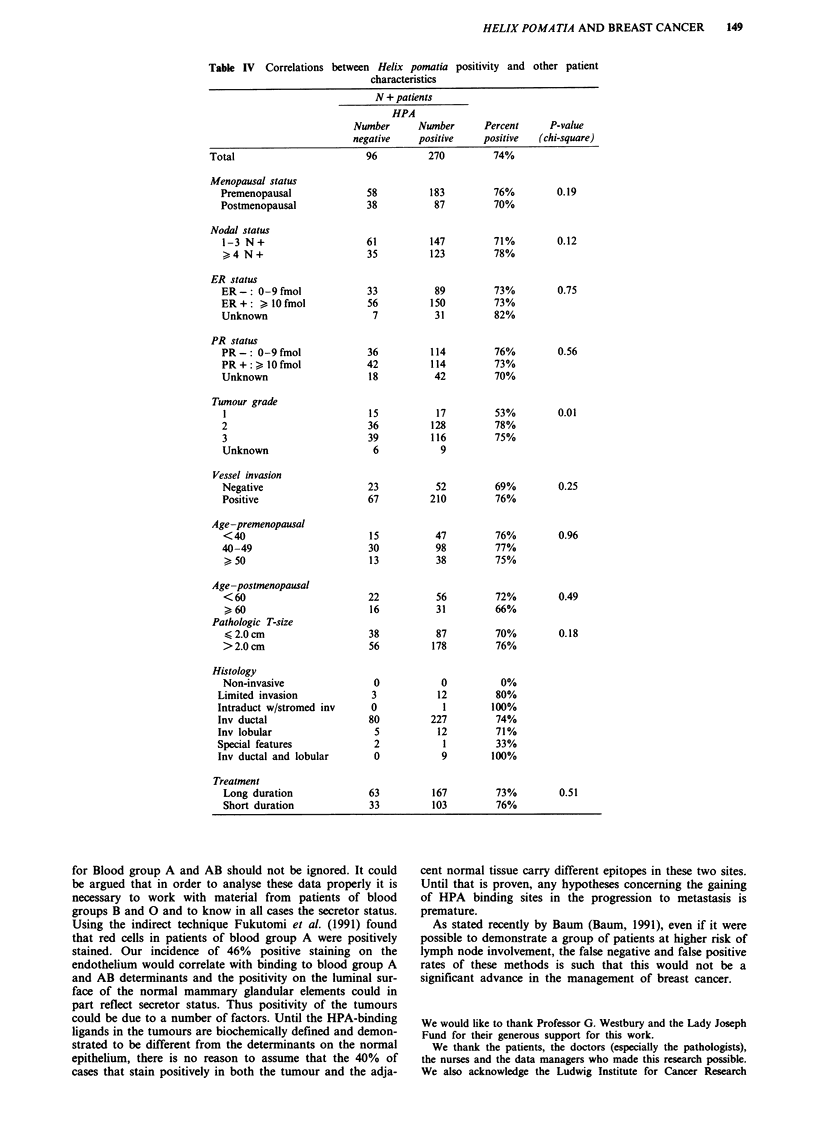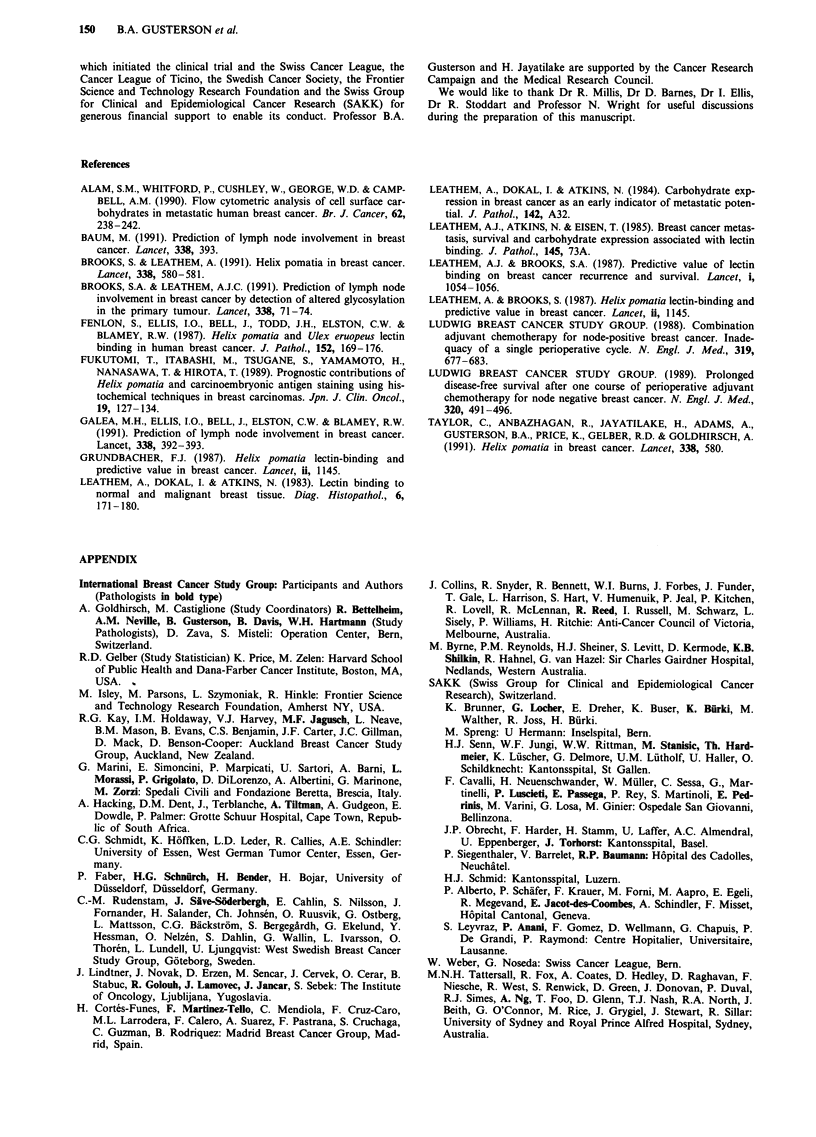# Prognostic value of Helix pomatia in breast cancer. International (Ludwig) Breast Cancer Study Group.

**DOI:** 10.1038/bjc.1993.303

**Published:** 1993-07

**Authors:** 

## Abstract

**Images:**


Br. J. Cancer (1993), 68, 146–150 © Macmillan Press Ltd., 1993

### Prognostic value of *Helix pomatia* in Breast Cancer

International (Ludwig) Breast Cancer Study Group
Writing Committee: B.A. Gusterson, C.W. Taylor, K.N. Price, R.D. Gelber, J. Säve-Söderbergh, R. Anbazhagan, H. Jayatilake, C.-M. Rudenstam, R. Reed, L. Morassi, P. Grigolato, M., Zorzi, R. Bettelheim, A.M. Neville, G.C. Hitchcock, M. Jagush, A. Tiltman, H.G. Schnürch, H. Bender, R. Golouh, J. Lamovec, J. Jancar, F. Martinez-Tello, K.B. Shilkin, G. Locher, K. Bürki, M. Stanisic, Th. Hardmeier, P. Luscieti, E. Passega, J. Torhorst, R.P. Baumann, E. Jacot-des-Coombes, P. Anani, A. Ng, M. Castiglione, H.-J. Senn & A. Goldhirsch. Other members of the Group are listed in the Appendix.

**Summary** Six hundred and eighty-four primary breast cancers from the International (Ludwig) Breast Cancer Study Group (IBCSG) were studied for *Helix pomatia* lectin (HPA) binding. There was a weak correlation between lymph node-positive and HPA positive ($P = 0.04$). In our series there was a large advantage in disease-free survival (DFS) and overall survival (OS) for node-negative patients ($P < 0.0001$ DFS and OS). However, there was no such advantage for HPA-negative patients ($P = 0.23$ DFS and $P = 0.32$ OS). We conclude that in this randomised patient group HPA is of no clinical predictive value.

Reports on the prognostic importance of *Helix pomatia* lectin binding in human breast cancer have been conflicting. In 1983 Leathem published that 14/14 normal breast specimens and 24/26 breast cancers stained positively with *Helix pomatia* lectin (Leathem *et al.*, 1983). This was followed by two meetings abstracts (Leathem *et al.*, 1984; Leathem *et al.*, 1985) from the same group that demonstrated a strong relationship between HPA binding and axillary lymph node metastases. In 1987 Fenlon *et al.*, in a series of 100 tumours, reported a significant correlation between HPA binding, tumour stage, local recurrence and survival (Fenlon *et al.*, 1987). The authors did not comment on menopausal status. In 1987 Leathem and Brooks reported that HPA positivity correlated with the time to first recurrence and with survival, but that this was only true for premenopausal women (Leathem & Brooks, 1987). This is in contrast to the results of Fukutomi *et al.* (1989) who found HPA positivity to be strongly correlated with poor survival, irrespective of menopausal status (Fukutomi *et al.*, 1989). In a recent report of 153 breast carcinomas using the same biotinylated lectin the significance had dropped to $P = 0.05$ (Fukutomi *et al.*, 1991). All of these earlier studies were carried out on small numbers of patients and there was a need for a careful analysis of large numbers of cases in different centres. Last year, Brooks and Leathem published a larger series of 373 cases (Brooks & Leathem, 1991). They found a strong correlation between HPA positivity and the presence of lymph node metastases. In this study there is no comment on whether this correlates with menopausal status and whether this is a completely new data set or an extension of their 179 cases previously reported (Leathem & Brooks, 1987). The study of Galea *et al.* (1991) on 459 cases could not confirm these data or their original observation (Fenlon *et al.*, 1987). On the basis of these conflicting results we carried out a pilot study on 363 cases randomised from the IBCSG (Ludwig) Trial V database. We found no correlation between HPA positivity and survival (Taylor *et al.*, 1991). In view of these findings we have enlarged the study to 684 cases from this same data set to re-analyse our HPA positivity in relation to all recorded clinical and pathological parameters. We report here a summary of our findings and some of the conflicting views in this area.

Correspondence: B.A. Gusterson, Institute of Cancer Research, Haddow Laboratories, Cotswold Road, Sutton, Surrey SM2 5NG, UK.
Received 7 May 1992; and in revised form 13 January 1993.

### Materials and methods

Cases were taken at random from 13 of the participating centres in the IBCSG Trial V. Details of this trial have been reported elsewhere (Ludwig Breast Cancer Study Group, 1988; Ludwig Breast Cancer Study Group, 1989). We have studied the association between HPA positivity and other prognostic factors and the effect of HPA binding on outcome. In view of the differences in the literature with staining techniques a pilot study was carried out by both the direct peroxidase conjugated method used by Galea (Galea *et al.*, 1991) and the indirect avidin-biotin techniques described by Fukutomi (Fukutomi *et al.*, 1989, 1991). Similar staining results were obtained for each and the positivity was inhibited by the appropriate sugar (N-acetyl-galactosamine), indicating that both methods produced specific staining. Figures 1 and 2 show similar fields of the same tumours stained by both methods. Both the tumour and the vascular endothelium show similar patterns of reactivity. Owing to the simplicity of the method all subsequent cases were stained using the direct method. All cases were scored according to the method described by Brooks & Leathem (1991), with all positive cases having either greater than 5% of the cells strongly positive to greater than 50% of the cells weakly positive. Samples were coded according to the patient randomisation number and all slides were reviewed independently by two pathologists (RA and BAG). In 10% of cases difficulty was encountered in using the scoring scheme and for these a consensus view was taken. All results were sent to the Biostatistics Center, Dana Farber Cancer Institute, where clinical correlations were studied.

### Results

It was noted that in a large proportion of cases there was staining of normal breast vascular endothelium and associated erythrocytes. On a representative sample of randomly selected cases (Table I) there was no clear correlation between positivity of tumour and normal tissues. Table IIA shows that there is a very weak correlation between *Helix pomatia* positivity and lymph node status and no correlation between HPA binding and either DFS or OS (Table IIB). In a detailed analysis of HPA binding and other patient characteristics no correlations were found with menopausal status, ER, PR, tumour grade, vessel invasion, histological type pathological tumour size or treatment groups (Tables III and IV). In addition, HPA does not predict a poorer prognosis in

**Figure 1** **a**, Photomicrograph of a breast carcinoma stained with the direct method for HPA immunoreactivity. (× 360). Note staining on vascular endothelium (arrows). **b**, The same tumour stained by the indirect method. Note the similar staining pattern of the tumour and the blood vessels. (× 360). Note staining on vascular endothelium (arrows).

**Table I** *Helix pomatia* lectin staining

| *Tumour* | *68 +* | | | | *32 −* | | | |
|---|---|---|---|---|---|---|---|---|
| Blood vessels | 33 + | | 35 − | | 13 + | | 19 − | |
| Normal breast | 18 ± | 15 − | 9 ± | 26 − | 4 ± | 9 − | 3 ± | 16 − |

+ Positive staining. − Negative staining. ± Positive and negative staining in different areas.

**Table IIA** *Helix pomatia* lectin binding by nodal status

| | *Number negative* | *Number positive* | *Percent positive* | *P-value (chi-square)* |
|---|---|---|---|---|
| Node negative | 106 | 212 | 67% | 0.04 |
| Node positive | 96 | 270 | 74% | (− vs. +) |
| 1–3 Nodes | 61 | 147 | 71% | |
| 4 + Nodes | 35 | 123 | 78% | |

**Table IIB** Six-year disease-free survival (DFS) and overall survival (OS) according to nodal status and *Helix pomatia* lectin positivity

| | *Pts* | *Relapsed* | *6-Year DFS ± e.* | *P-value* | *Deaths* | *6-Year OS ± e* | *P-value* |
|---|---|---|---|---|---|---|---|
| Node negative | 318 | 105 | 66 ± 3 | <0.0001 | 55 | 83 ± 2 | <0.00 |
| Node positive | 366 | 197 | 47 ± 3 | | 131 | 62 ± 3 | 01 |
| HPA − | 202 | 83 | 59 ± 4 | 0.23 | 50 | 76 ± 3 | 0.32 |
| HPA + | 482 | 219 | 55 ± 2 | | 136 | 70 ± 2 | |

any of the above sub-groups for DFS or OS. In the lymph node positive group there was a correlation of HPA with increased tumour grade, but there were only 32 cases in the Grade 1 category, making this result of doubtful significance.

### Discussion

This detailed analysis has failed to confirm some of the previous published data. As we have a similar percentage of positive cases and have demonstrated specificity of staining, it is difficult to explain away the differences methodologically. As Brooks and Leathem have recently stated that the differences seen in previously published data can be explained on the basis of staining techniques (Brooks & Leathem, 1991), a number of points need to be addressed here. Firstly, Brooks and Leathem state that they use an indirect method because this provides a clinical correlation not seen with direct HPA-peroxidase conjugate technique. We found no difference in staining pattern between the avidin-biotin and the direct method in our pilot study. It can not be excluded that using other sources of HPA and staining methods a correlation with survival would be observed, but it can be concluded that HPA binding as demonstrated

**Table III** Correlations between *Helix pomatia* (HPA) positivity and other patient characteristics

| | *N – patients* | | | |
|---|---|---|---|---|
| | *HPA* | | | |
| | *Number negative* | *Number positive* | *Percent positive* | *P-value (chi-square)* |
| Total | 106 | 212 | 67% | |
| *Menopausal status* | | | | |
| Premenopausal | 62 | 115 | 65% | 0.47 |
| Postmenopausal | 44 | 97 | 69% | |
| *ER status* | | | | |
| ER – : 0–9 fmol | 42 | 76 | 64% | 0.54[a] |
| ER + : ⩾ 10 fmol | 54 | 114 | 68% | |
| Unknown | 10 | 22 | 69% | |
| *PR status* | | | | |
| PR – : 0–9 fmol | 51 | 95 | 65% | 0.64[a] |
| PR + : ⩾ 10 fmol | 37 | 78 | 68% | |
| Unknown | 18 | 39 | 68% | |
| *Tumour grade* | | | | |
| 1 | 13 | 29 | 69% | 0.14[a] |
| 2 | 38 | 99 | 72% | |
| 3 | 45 | 69 | 61% | |
| Unknown | 10 | 15 | 60% | |
| *Vessel invasion* | | | | |
| Negative | 52 | 103 | 66% | 0.94[a] |
| Positive | 49 | 99 | 67% | |
| Unknown | 5 | 10 | 67% | |
| *Age–premenopausal* | | | | |
| <40 | 14 | 22 | 61% | 0.36 |
| 40–49 | 31 | 70 | 69% | |
| ⩾ 50 | 17 | 23 | 58% | |
| *Age–postmenopausal* | | | | |
| <60 | 25 | 47 | 65% | 0.36 |
| ⩾ 60 | 19 | 50 | 72% | |
| *Histology* | | | | |
| Non-invasive | 1 | 2 | 67% | |
| Limited invasion | 5 | 14 | 74% | |
| Intraductal w/stomal inv | 4 | 4 | 50% | |
| Inv ductal | 78 | 147 | 65% | |
| Inv lobular | 9 | 21 | 70% | |
| Special features | 5 | 9 | 64% | |
| Inv ductal and lobular | 0 | 6 | 100% | |
| *Pathologic T-size* | | | | |
| ⩽ 2.0 cm | 49 | 100 | 67% | 0.72 |
| >2.0 cm | 55 | 103 | 65% | |
| *Treatment* | | | | |
| PeCT | 75 | 133 | 64% | 0.16 |
| No PeCT | 31 | 79 | 72% | |

[a]*P*-value calculations do not include 'unknown' categories.

here with the appropriate sugar controls does not. The avidin-biotin-peroxidase method was used by Fukutomi *et al.* (1989) in the paper quoted by Brooks in support of their own findings. In the flow cytometry paper of Alam *et al.* (1990), that also found a positive correlation between HPA positivity and lymph node involvement, the authors used a direct technique. They, however, state that they selected a cut off when related to grade and lymph node involvement of 20% of cells positive as this was the most informative (Alam *et al.*, 1990). It is difficult therefore to draw any conclusions from this publication until it is repeated by other groups with a larger data set.

We do not rule out the possibility that two staining methods with a lectin may give a different result dependent upon the cut off used for positivity. The difficulty is that with any study there is a statistical chance finding of a significant correlation between any given parameter and any selected cut off point. We must conclude that there is no consistent data to strongly support the view that the localisation of HPA epitopes is of clinical significance. This issue will not be resolved on the basis of staining procedures. As discussed by Brooks and Leathem (1991), the binding of lectins is very complex and poorly understood. It is therefore essential that the reactive epitopes are properly characterised and more suitable antibodies with defined specificity made available. We await with interest the biochemical evidence for, and characterisation of, the glycoconjugate that Leathem *et al.*, first identified immunocytochemically in 1983.

The lack of correlation between staining of tumour and normal tissues is very important because, although addressed in part by correspondence in the Lancet between Leathem (Leathem & Brooks, 1987) and Grundbacher (Grundbacher *et al.*, 1987), the possible confounding effects of any analyses by secretor status and the binding ability of *Helix pomatia*

**Table IV** Correlations between *Helix pomatia* positivity and other patient characteristics

| | *N + patients* | | | |
|---|---|---|---|---|
| | *HPA* | | | |
| | *Number negative* | *Number positive* | *Percent positive* | *P-value (chi-square)* |
| Total | 96 | 270 | 74% | |
| *Menopausal status* | | | | |
| Premenopausal | 58 | 183 | 76% | 0.19 |
| Postmenopausal | 38 | 87 | 70% | |
| *Nodal status* | | | | |
| 1–3 N+ | 61 | 147 | 71% | 0.12 |
| ⩾4 N+ | 35 | 123 | 78% | |
| *ER status* | | | | |
| ER – : 0–9 fmol | 33 | 89 | 73% | 0.75 |
| ER + : ⩾ 10 fmol | 56 | 150 | 73% | |
| Unknown | 7 | 31 | 82% | |
| *PR status* | | | | |
| PR – : 0–9 fmol | 36 | 114 | 76% | 0.56 |
| PR + : ⩾ 10 fmol | 42 | 114 | 73% | |
| Unknown | 18 | 42 | 70% | |
| *Tumour grade* | | | | |
| 1 | 15 | 17 | 53% | 0.01 |
| 2 | 36 | 128 | 78% | |
| 3 | 39 | 116 | 75% | |
| Unknown | 6 | 9 | | |
| *Vessel invasion* | | | | |
| Negative | 23 | 52 | 69% | 0.25 |
| Positive | 67 | 210 | 76% | |
| *Age–premenopausal* | | | | |
| <40 | 15 | 47 | 76% | 0.96 |
| 40–49 | 30 | 98 | 77% | |
| ⩾ 50 | 13 | 38 | 75% | |
| *Age–postmenopausal* | | | | |
| <60 | 22 | 56 | 72% | 0.49 |
| ⩾ 60 | 16 | 31 | 66% | |
| *Pathologic T-size* | | | | |
| ⩽ 2.0 cm | 38 | 87 | 70% | 0.18 |
| >2.0 cm | 56 | 178 | 76% | |
| *Histology* | | | | |
| Non-invasive | 0 | 0 | 0% | |
| Limited invasion | 3 | 12 | 80% | |
| Intraduct w/stromed inv | 0 | 1 | 100% | |
| Inv ductal | 80 | 227 | 74% | |
| Inv lobular | 5 | 12 | 71% | |
| Special features | 2 | 1 | 33% | |
| Inv ductal and lobular | 0 | 9 | 100% | |
| *Treatment* | | | | |
| Long duration | 63 | 167 | 73% | 0.51 |
| Short duration | 33 | 103 | 76% | |

for Blood group A and AB should not be ignored. It could be argued that in order to analyse these data properly it is necessary to work with material from patients of blood groups B and O and to know in all cases the secretor status. Using the indirect technique Fukutomi *et al.* (1991) found that red cells in patients of blood group A were positively stained. Our incidence of 46% positive staining on the endothelium would correlate with binding to blood group A and AB determinants and the positivity on the luminal surface of the normal mammary glandular elements could in part reflect secretor status. Thus positivity of the tumours could be due to a number of factors. Until the HPA-binding ligands in the tumours are biochemically defined and demonstrated to be different from the determinants on the normal epithelium, there is no reason to assume that the 40% of cases that stain positively in both the tumour and the adjacent normal tissue carry different epitopes in these two sites. Until that is proven, any hypotheses concerning the gaining of HPA binding sites in the progression to metastasis is premature.

As stated recently by Baum (Baum, 1991), even if it were possible to demonstrate a group of patients at higher risk of lymph node involvement, the false negative and false positive rates of these methods is such that this would not be a significant advance in the management of breast cancer.

We would like to thank Professor G. Westbury and the Lady Joseph Fund for their generous support for this work.

We thank the patients, the doctors (especially the pathologists), the nurses and the data managers who made this research possible. We also acknowledge the Ludwig Institute for Cancer Research which initiated the clinical trial and the Swiss Cancer League, the Cancer League of Ticino, the Swedish Cancer Society, the Frontier Science and Technology Research Foundation and the Swiss Group for Clinical and Epidemiological Cancer Research (SAKK) for generous financial support to enable its conduct. Professor B.A. Gusterson and H. Jayatilake are supported by the Cancer Research Campaign and the Medical Research Council.

We would like to thank Dr R. Millis, Dr D. Barnes, Dr I. Ellis, Dr R. Stoddart and Professor N. Wright for useful discussions during the preparation of this manuscript.

### References

ALAM, S.M., WHITFORD, P., CUSHLEY, W., GEORGE, W.D. & CAMPBELL, A.M. (1990). Flow cytometric analysis of cell surface carbohydrates in metastatic human breast cancer. *Br. J. Cancer*, **62**, 238–242.

BAUM, M. (1991). Prediction of lymph node involvement in breast cancer. *Lancet*, **338**, 393.

BROOKS, S. & LEATHEM, A. (1991). Helix pomatia in breast cancer. *Lancet*, **338**, 580–581.

BROOKS, S.A. & LEATHEM, A.J.C. (1991). Prediction of lymph node involvement in breast cancer by detection of altered glycosylation in the primary tumour. *Lancet*, **338**, 71–74.

FENLON, S., ELLIS, I.O., BELL, J., TODD, J.H., ELSTON, C.W. & BLAMEY, R.W. (1987). *Helix pomatia* and *Ulex eruopeus* lectin binding in human breast cancer. *J. Pathol.*, **152**, 169–176.

FUKUTOMI, T., ITABASHI, M., TSUGANE, S., YAMAMOTO, H., NANASAWA, T. & HIROTA, T. (1989). Prognostic contributions of *Helix pomatia* and carcinoembryonic antigen staining using histochemical techniques in breast carcinomas. *Jpn. J. Clin. Oncol.*, **19**, 127–134.

GALEA, M.H., ELLIS, I.O., BELL, J., ELSTON, C.W. & BLAMEY, R.W. (1991). Prediction of lymph node involvement in breast cancer. Lancet, **338**, 392–393.

GRUNDBACHER, F.J. (1987). *Helix pomatia* lectin-binding and predictive value in breast cancer. *Lancet*, **ii**, 1145.

LEATHEM, A., DOKAL, I. & ATKINS, N. (1983). Lectin binding to normal and malignant breast tissue. *Diag. Histopathol.*, **6**, 171–180.

LEATHEM, A., DOKAL, I. & ATKINS, N. (1984). Carbohydrate expression in breast cancer as an early indicator of metastatic potential. *J. Pathol.*, **142**, A32.

LEATHEM, A.J., ATKINS, N. & EISEN, T. (1985). Breast cancer metastasis, survival and carbohydrate expression associated with lectin binding. *J. Pathol.*, **145**, 73A.

LEATHEM, A.J. & BROOKS, S.A. (1987). Predictive value of lectin binding on breast cancer recurrence and survival. *Lancet*, **i**, 1054–1056.

LEATHEM, A. & BROOKS, S. (1987). *Helix pomatia* lectin-binding and predictive value in breast cancer. *Lancet*, **ii**, 1145.

LUDWIG BREAST CANCER STUDY GROUP. (1988). Combination adjuvant chemotherapy for node-positive breast cancer. Inadequacy of a single perioperative cycle. *N. Engl. J. Med.*, **319**, 677–683.

LUDWIG BREAST CANCER STUDY GROUP. (1989). Prolonged disease-free survival after one course of perioperative adjuvant chemotherapy for node negative breast cancer. *N. Engl. J. Med.*, **320**, 491–496.

TAYLOR, C., ANBAZHAGAN, R., JAYATILAKE, H., ADAMS, A., GUSTERSON, B.A., PRICE, K., GELBER, R.D. & GOLDHIRSCH, A. (1991). *Helix pomatia* in breast cancer. *Lancet*, **338**, 580.

### APPENDIX

**International Breast Cancer Study Group:** Participants and Authors (Pathologists **in bold type**)

A. Goldhirsch, M. Castiglione (Study Coordinators) **R. Bettelheim, A.M. Neville, B. Gusterson, B. Davis, W.H. Hartmann** (Study Pathologists), D. Zava, S. Misteli: Operation Center, Bern, Switzerland.

R.D. Gelber (Study Statistician) K. Price, M. Zelen: Harvard School of Public Health and Dana-Farber Cancer Institute, Boston, MA, USA.

M. Isley, M. Parsons, L. Szymoniak, R. Hinkle: Frontier Science and Technology Research Foundation, Amherst NY, USA.

R.G. Kay, I.M. Holdaway, V.J. Harvey, **M.F. Jagusch**, L. Neave, B.M. Mason, B. Evans, C.S. Benjamin, J.F. Carter, J.C. Gillman, D. Mack, D. Benson-Cooper: Auckland Breast Cancer Study Group, Auckland, New Zealand.

G. Marini, E. Simoncini, P. Marpicati, U. Sartori, A. Barni, **L. Morassi, P. Grigolato**, D. DiLorenzo, A. Albertini, G. Marinone, **M. Zorzi**: Spedali Civili and Fondazione Beretta, Brescia, Italy.

A. Hacking, D.M. Dent, J., Terblanche, **A. Tiltman**, A. Gudgeon, E. Dowdle, P. Palmer: Grotte Schuur Hospital, Cape Town, Republic of South Africa.

C.G. Schmidt, K. Höffken, L.D. Leder, R. Callies, A.E. Schindler: University of Essen, West German Tumor Center, Essen, Germany.

P. Faber, **H.G. Schnürch, H. Bender**, H. Bojar, University of Düsseldorf, Düsseldorf, Germany.

C.-M. Rudenstam, **J. Säve-Söderbergh**, E. Cahlin, S. Nilsson, J. Fornander, H. Salander, Ch. Johnsén, O. Ruusvik, G. Ostberg, L. Mattsson, C.G. Bäckström, S. Bergegårdh, G. Ekelund, Y. Hessman, O. Nelzén, S. Dahlin, G. Wallin, L. Ivarsson, O. Thorén, L. Lundell, U. Ljungqvist: West Swedish Breast Cancer Study Group, Göteborg, Sweden.

J. Lindtner, J. Novak, D. Erzen, M. Sencar, J. Cervek, O. Cerar, B. Stabuc, **R. Golouh, J. Lamovec, J. Jancar**, S. Sebek: The Institute of Oncology, Ljubljana, Yugoslavia.

H. Cortès-Funes, **F. Martinez-Tello**, C. Mendiola, F. Cruz-Caro, M.L. Larrodera, F. Calero, A. Suarez, F. Pastrana, S. Cruchaga, C. Guzman, B. Rodriquez: Madrid Breast Cancer Group, Madrid, Spain.

J. Collins, R. Snyder, R. Bennett, W.I. Burns, J. Forbes, J. Funder, T. Gale, L. Harrison, S. Hart, V. Humenuik, P. Jeal, P. Kitchen, R. Lovell, R. McLennan, **R. Reed**, I. Russell, M. Schwarz, L. Sisely, P. Williams, H. Ritchie: Anti-Cancer Council of Victoria, Melbourne, Australia.

M. Byrne, P.M. Reynolds, H.J. Sheiner, S. Levitt, D. Kermode, **K.B. Shilkin**, R. Hahnel, G. van Hazel: Sir Charles Gairdner Hospital, Nedlands, Western Australia.

SAKK (Swiss Group for Clinical and Epidemiological Cancer Research), Switzerland.

K. Brunner, **G. Locher**, E. Dreher, K. Buser, **K. Bürki**, M. Walther, R. Joss, H. Bürki.

M. Spreng: U Hermann: Inselspital, Bern.

H.J. Senn, W.F. Jungi, W.W. Rittman, **M. Stanisic, Th. Hardmeier**, K. Lüscher, G. Delmore, U.M. Lüthölf, U. Haller, O. Schildknecht: Kantonsspital, St Gallen.

F. Cavalli, H. Neuenschwander, W. Müller, C. Sessa, G., Martinelli, **P. Luscieti, E. Passega**, P. Rey, S. Martinoli, **E. Pedrinis**, M. Varini, G. Losa, M. Ginier: Ospedale San Giovanni, Bellinzona.

J.P. Obrecht, F. Harder, H. Stamm, U, Laffer, A.C. Almendral, U. Eppenberger, **J. Torhorst:** Kantonsspital, Basel.

P. Siegenthaler, V. Barrelet, **R.P. Baumann:** Hôpital des Cadolles, Neuchâtel.

H.J. Schmid: Kantonsspital, Luzern.

P. Alberto, P. Schäfer, F. Krauer, M. Forni, M. Aapro, E. Egeli, R. Megevand, **E. Jacot-des-Coombes**, A. Schindler, F. Misset, Hôpital Cantonal, Geneva.

S. Leyvraz, **P. Anani**, F. Gomez, D. Wellmann, G. Chapuis, P. De Grandi, P. Raymond: Centre Hopitalier, Universitaire, Lausanne.

W. Weber, G. Noseda: Swiss Cancer League, Bern.

M.N.H. Tattersall, R. Fox, A. Coates, D. Hedley, D. Raghavan, F. Niesche, R. West, S. Renwick, D. Green, J. Donovan, P. Duval, R.J. Simes, **A. Ng**, T. Foo, D. Glenn, T.J. Nash, R.A. North, J. Beith, G. O'Connor, M. Rice, J. Grygiel, J. Stewart, R. Sillar: University of Sydney and Royal Prince Alfred Hospital, Sydney, Australia.